# Chromosomal instability (CIN) triggers immune evasion and metastatic potential in cancer through rewired STING signalling

**DOI:** 10.1186/s43556-023-00166-8

**Published:** 2024-01-23

**Authors:** Mrinal K. Ghosh, Srija Roy

**Affiliations:** Cancer Biology and Inflammatory Disorder Division, Council of Scientific and Industrial Research-Indian Institute of Chemical Biology (CSIR-IICB), TRUE Campus, CN-6, Sector–V, Salt Lake, Kolkata-700091 & 4, Raja S.C. Mullick Road, Jadavpur, Kolkata, 700032 India

Recently, an article titled “Non-cell-autonomous cancer progression from chromosomal instability” was published in Nature, Aug 2023. In this study, Li *et. al.* elaborated a dependency of immune cells enrichment in the tumour microenvironment (TME) on chromosomal instability (CIN) in cancer cells [1]. This insightful research sheds light on the potential to selectively target downstream molecules of CIN in a context-specific approach to achieve maximum effectiveness in reducing metastasis in aggressive cancers such as triple negative breast cancer (TNBC), colorectal carcinoma, and melanoma. The above-mentioned context is acute or chronic activation of cGAS-STING (cyclic GMP-AMP synthase-Stimulator of Interferon Genes) innate immune signalling pathway in response to CIN, which determines whether the cell mounts immune-activating phenotype or undergoes endoplasmic reticulum (ER) stress, enabling it to evade immune surveillance and metastasize (Fig. 1).

**Fig. 1:**
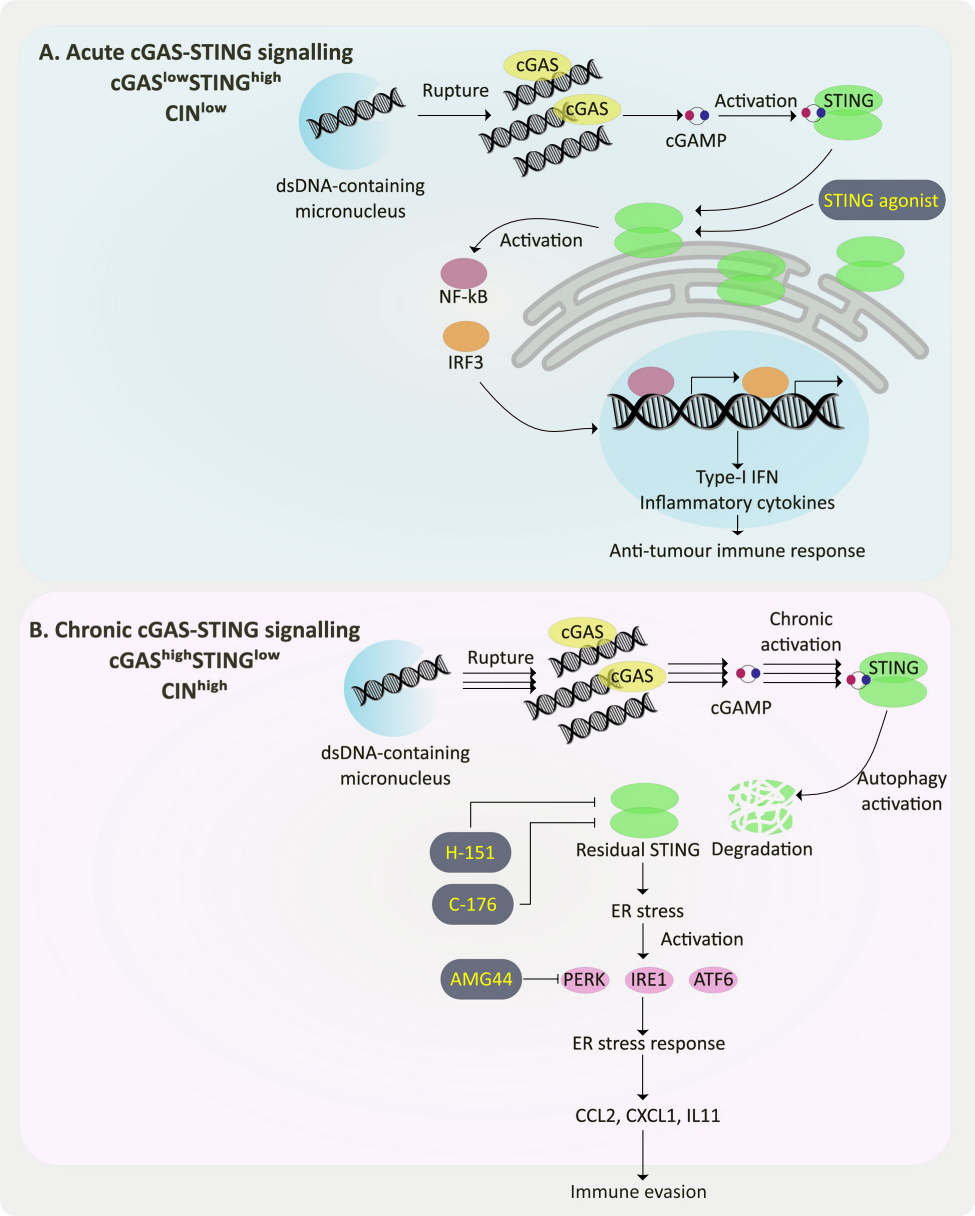
Chromosomal instability (CIN) leads to double strand DNA (dsDNA)-mediated cGAS-STING pathway activation. Context-dependent functional outcome of STING leads to either interferon (IFN) response-mediated anti-tumour immunity or ER stress response-mediated immune evasion and subsequent distant metastasis. Drugs (agonist/inhibitors) enhance treatment efficacy depending on tumour subtypes. **a** Acute cGAS-STING signalling promotes anti-tumour immunity through activation of transcription factors like NF-κB and IRF3. **b** Chronic cGAS-STING signalling leads to immune evasion through ER stress response

Proposed by Theodor Boveri in 1914, CIN remains a relatively underexplored hallmark of cancer. This condition arises from abnormal chromosomal segregation during anaphase, leading to numerical (aneuploidy) and structural abnormalities. The mis-segregated chromosomes form micronuclei, dispersing into the cytoplasm during interphase. Notably, genomic double-stranded DNA (dsDNA)-containing micronuclei are a distinctive feature of CIN. These micronuclei are prone to rupture within the cytoplasm, triggering the anti-viral cGAS-STING signalling pathway. Mechanistically, cytoplasmic dsDNA activates cGAS-mediated cGAMP production, which in turn activates STING resulting in the latter’s localization at the ER membrane and subsequent transcriptional activation of pro-inflammatory interferon-stimulated genes (ISGs). On the other hand, immune evasion is also a well-recognized hallmark of cancer, marked by a notable decrease in anti-tumour immune cells within the TME. Therefore, the paradox arises from the question of how cancer cells manage to maintain elevated levels of CIN while concurrently evading immune surveillance [2].

In this paper, the researchers have attempted to address the above question through genetic manipulation, single-cell RNA sequencing (scRNA-Seq) and a newly developed tool ContactTracing. First, they genetically engineered CIN^low^ and CIN^high^ TNBC cell lines by expressing CIN-reducing genes and their dominant-negative counterparts, respectively. Then they transplanted these engineered cells and found that the number of surface lung metastases were significantly reduced in CIN^low^ versus CIN^high^ tumours in immune-competent but not in immune-compromised mice. This immune-dependency of CIN-induced metastatic potential was also observed in *Cgas*/*Sting1*-depleted or knocked-down CIN^high^ cells. Next, they utilized scRNA-Seq to elucidate that CIN^high^ tumours harvested from immune-competent mice models were enriched in anti-inflammatory macrophages, granulocytic myeloid-derived suppressor cells (Gr-MDSCs), and dysfunctional T cells. CIN inhibition or *Cgas/Sting1* knockout resulted in CD8^+^ cytotoxic T cell infiltration in such tumours. Conversely, CIN^low^ tumours were associated with pro-inflammatory macrophages, activated dendritic cells and CD4^+^ helper T cells. Furthermore, they developed a highly sophisticated and validated tool called ContactTracing to analyse condition-dependent cell–cell interactions from scRNA-Seq data. Through ContactTracing, they observed that CIN^high^ tumours with immunosuppressive TME, expressed ER stress response cytokines like *Ccl2*, *Cxcl1*, and *Il11* in addition to NF-κB and IL6/Jak/Stat3 pathways. In contrast, CIN^low^ tumours were primarily associated with interferon (IFN) responses.

The ER stress response was dependent on STING expression as reduced response was associated with *Sting1*-knockout CIN^high^ tumours even in the presence of ER stress inducer, tunicamycin (TM). However, expression of ER stress cytokines in *Sting1*-knockout CIN^high^ tumours resulted in enhanced metastasis. Consequently, CRISPR-Cas9-mediated knockout of ER stress sensors, IRE1α, PERK or ATF6, resulted in decreased surface lung metastases. Likewise, AMG44 (a selective PERK inhibitor) treatment of CIN^high^ tumours led to increased CD8^+^ T cell and natural killer cell infiltration, in an immunocompetence-dependent manner. Additionally, STING inhibition by C-176 or H-151 reduced inflammation, epithelial-mesenchymal transition (EMT), ER stress response and increased survival of immune-competent mice. However, reduced IFN response in CIN^high^ tumours as compared to CIN^low^ tumours indicates context-dependent expression level alteration of STING. Li and colleagues defined this context-dependency of cellular responses on STING levels using a tractable model system. Existence of a tachyphylactic phenomenon, where in CIN^high^ cells chronic activation of STING leading to autophagy-mediated STING degradation was found, resulting in reduced IFN signalling and increased ER stress response.

This impressive study has unveiled the presence of two distinct tumour subtypes within human TNBC samples, namely cGAS^low^STING^high^ and cGAS^high^STING^low^ associated with acute and chronic CIN, respectively. Notably, the former subtype demonstrated a more favorable prognosis when compared to the latter. Consequently, the researchers propose patient-level stratification into CIN^high^ and CIN^low^ categories based on the molecular abundances of cGAS and STING to enhance treatment outcomes.

In summary, this study has tackled the crucial question of the relationship between the cancer hallmark CIN and its downstream effector, STING, both of which have opposing effects on tumour progression. The researchers have argued that in cancer, CIN leads to a rewiring of the cGAS-STING pathway, resulting in an induction of the ER stress response and the creation of an immunosuppressive TME. In conclusion, patients with a cGAS^low^STING^high^ profile are expected to benefit from STING activators because of their ability to trigger an interferon (IFN) response, while patients with a cGAS^high^STING^low^ profile are more likely to respond positively to STING inhibitors, especially when used in combination with ER stress inhibitors [3–5].

## Data Availability

All information derived for the preparation of the manuscript is based on the data available in the cited research articles.

## References

[1] Li J, Hubisz MJ, Earlie EM, Duran MA, Hong C, Varela AA (2023). Non-cell-autonomous cancer progression from chromosomal instability. Nature..

[2] Bakhoum SF, Cantley LC (2018). The multifaceted role of chromosomal instability in cancer and its microenvironment. Cell..

[3] Guerini D (2022). STING agonists/antagonists: their potential as therapeutics and future developments. Cells..

[4] Wu YT, Fang Y, Wei Q, Shi H, Tan H, Deng Y, et al. Tumor-targeted delivery of a STING agonist improves cancer immunotherapy. Proc Natl Acad Sci USA. 2022;119(49):e2214278119. 10.1073/pnas.2214278119. 10.1073/pnas.2214278119 PMC9894229 36442099

[5] Xu D, Liu Z, Liang MX, Fei YJ, Zhang W, Wu Y (2022). Endoplasmic reticulum stress targeted therapy for breast cancer. Cell Commun Signal CCS..

